# Supplementary material for: c-D-index at day 11 can predict febrile neutropenia during chemotherapy in acute myeloid leukemia
Source: PLoS One. 2022 Mar 17;17(3):e0263623. doi: 10.1371/journal.pone.0263623 (PMC8929597; doi:10.1371/journal.pone.0263623)
Supplement: Attachment — Submitted filename: Reviewer comment.docx [file pone.0263623.s002.docx]

**c-D-index can predict febrile neutropenia during chemotherapy in acute myeloid leukemia**

İntroduction:

- It is better to explain population as: ‘adult patients’.
- Definition of febrile neutropenia needs correction and	reference.
- ‘We obtained informed consent to participate from all patients.’ .Due to retrospective nature of the study , it seems hard to take informed consent from all patients.

In results section:

- Was c-D-index different according to organism identified as the cause of febrile neutropenia?
- It is better to give values of c-D-index according to cultured gram positive / gram negative organisms.

‘We comprehensively categorized the patients’ clinical symptoms

into six focuses of infection according to clinical manifestations and culture results: oral

cavity (stomatitis, toothache, and gingivitis), gastrointestinal (nausea, vomiting, diarrhea, and

abdominal pain), respiratory tract (cough, sputum, dyspnea, and chest pain), skin (rash,

swelling, flare, and painful skin, including in the device insertion area), bloodstream (blood

culture positive but lacking in clinical manifestations), and unknown origin (blood culture

negative and lacking in clinical manifestations or the coexistence of multiple clinical

manifestations that would differentiate other diagnoses).’

- Was c-D-index different accordinf to focuses of infection?
- What about IFI? Was there any IFI cases in group?

In discussion section

-‘Portugal *et al*. reported a c-D-index cutoff value of ≥ 5500 for sensitive prediction of IFI.’Reference is needed.

-Discussion is inadequate.
